# Induction of puberty *vs.* induction of ovulation using steroid hormones in beef heifers: a comprehensive review

**DOI:** 10.1590/1984-3143-AR2024-0072

**Published:** 2024-09-09

**Authors:** Roberto Sartori, Rodrigo Lemos Olivieri Rodrigues Alves, Ana Luíza Müller Lopes

**Affiliations:** 1 Escola Superior de Agricultura “Luiz de Queiroz” (ESALQ), Universidade de São Paulo (USP), Piracicaba, SP, Brasil

**Keywords:** fertility, ovulation, puberty, reproductive performance

## Abstract

This review elucidates the physiological and endocrinological processes intrinsic to puberty and ovulation induction protocols in *Bos indicus* and *Bos taurus* beef heifers. Puberty is a complex physiological event involving gonadotropic and metabolic changes that lead to sexual maturity, first ovulation, and regular reproductive cycles, enabling females to reproduce. Exposure to progesterone-based hormonal protocols, with or without additional hormones, can reduce the age at first ovulation and improve sexual maturity through stimuli in the hypothalamus-pituitary-gonadal (HPG) axis and uterine development. However, inducing puberty differs from inducing ovulation, as it does not ensure the heifer will continue cycling or be ready to establish and maintain pregnancy after hormonal exposure. Regardless of the pharmacological basis, studies consistently report that beef heifers that had a corpus luteum (CL) prior to the timed-artificial insemination (TAI) protocol, have greater expression of estrus in response to synchronization and greater pregnancy per AI compared to heifers without a CL. The combination of P4 and E2 significantly impacts uterine development, increasing reproductive efficiency. Exposure to P4 causes a positive effect on inducing ovulation. However, studies indicate that the addition of E2 esters at the time of P4 device removal increases the ovulation rate. In general, the studies showed that fertility varied according to the type of the ovulation induction protocol used, but with inconsistent results. Although ovulation induction protocols are strategic tools to accelerate sexual maturity, a holistic view of the entire system is extremely important, combining integration with genetics and nutrition to enhance the reproductive outcomes of beef heifers. Future research is needed to understand and refine these protocols, driving the efficiency of beef cattle production systems.

## Introduction

In cow-calf operations, heifers are recognized as having the highest genetic merit compared to multiparous cows, as they benefit from recent advances in genetic selection and reproductive technologies (Wathes et al., 2014). This drives considerable research interest in their physiology. The timing of conception and age at calving, plays a crucial role in operational reproductive and productive efficiency because it is directly linked to future productivity (Brumatti et al., 2011; Núñez-Dominguez et al., 1991), leading to extensive efforts aimed at optimizing this process. However, many farms experience less than optimal efficiency, characterized by heifers conceiving and calving at later ages (Gunski et al., 2001; Nogueira, 2004). Consequently, the search for strategies to induce puberty at an earlier age and/or the beginning of reproductive activity in these females, in addition to increasing reproductive efficiency, is extremely important.

The onset of reproductive life in female cattle is not an isolated event and depends on morphophysiological changes involving maturation of the reproductive tract and the HPG axis (Day et al., 1987; Moran et al., 1989). Genetic and nutritional factors directly influence the age at puberty (Ferraz et al., 2018), as well as the ability of females to conceive and maintain pregnancy. Alongside this, hormonal protocols assist in advancing the occurrence of the first ovulation, but do not necessarily induce puberty in females, as many heifers that ovulate in response to the protocols are not yet physiologically prepared to maintain cyclic activity and be fit for reproductive purposes. In this context, the aim of this section of the manuscript is to bring and discuss the main advances regarding ovulation induction strategies carried out prior to the timed-AI (TAI) protocol, in *Bos indicus* and *Bos taurus* beef heifers

## Induction of puberty vs. induction of ovulation

Puberty is a complex physiological event marked by gonadotropic and metabolic changes. It marks the onset of sexual maturity, the first ovulation, and the establishment of regular reproductive cycles, enabling females to reproduce (Cardoso et al., 2020; D’Occhio et al., 2019; Day et al., 1984). A pre-pubertal condition is marked by a low frequency of luteinizing hormone (LH) pulses, causing dominant follicles not to grow enough to generate a peak of estradiol (E2), resulting in a lack of pre-ovulatory peak of gonadotropin-releasing hormone (GnRH)/LH (Day et al., 1984, 1987).

During the prepubertal phase, E2 produces negative feedback at the hypothalamus, mainly in the tonic center, and anterior pituitary, reducing the pulse frequency and amplitude of GnRH/LH (Day et al., 1987; Schillo et al., 1982). As puberty approaches, there is an increase in LH pulsatility, resulting in stimulation of final follicular growth, leading to a peak in E2, followed by a peak in GnRH/LH and ovulation. The reduction of negative feedback produced by E2 occurs due to a decrease in its receptors in the hypothalamus, resulting in an increase in GnRH and LH pulse frequency (Day et al., 1987). However, GnRH neurons do not possess alpha receptors for E2, indicating that the negative feedback of E2 does not occur directly at the level of GnRH neurons, but rather through a neuronal network. According to the literature, neurons called KNDy (kisspeptin – KP, neurokinin B – NKB, and dynorphin – DYN), situated in the arcuate nucleus of hypothalamus, seem to mediate the effects of E2 on GnRH response through the release of KP (Nestor et al., 2023). Apparently, NKB stimulates KP neurons, while DYN is responsible for inhibiting the action of this neuronal network (Cardoso et al., 2020, 2018). Moreover, KP neurons are situated in the preoptic area of the hypothalamus and stimulates GnRH release (Scott et al., 2018).

The mechanism by which P4 acts on the HPG axis in prepubertal bovine females is still not fully elucidated. Day and Anderson (1998) suggested that exposure to P4 decreases the amount of E2 receptors in hypothalamic neurons, reducing the negative feedback of E2 on GnRH and LH secretion. Exposing bovine females to exogenous sources of P4 in low concentrations for a determined period stimulates the pulsatility of GnRH and LH, resulting in greater follicular growth and sufficient E2 production to generate a preovulatory peak of GnRH and LH, culminating in ovulation after the removal of the P4 source (Anderson et al., 1996; Imwalle et al., 1998; Rasby et al., 1998; Claro et al., 2010). In a study conducted by our group (Alves et al., 2023a), prepubertal Nelore heifers were randomly assigned to either low circulating P4 concentrations using a 1 g implant for 14 d (LP4 group) or a control group with a placebo implant without P4 (CON group). The IVD were maintained for 22 d. The LP4 group exhibited a higher average level of circulating P4 (1.1 ± 0.1 vs. 0.3 ± 0.1 ng/mL), a larger average dominant follicle diameter (14.4 ± 0.6 vs. 12.2 ± 0.5 mm), and a longer interval between follicular waves (8.7 ± 0.5 vs. 6.6 ± 0.5 d) compared to the CON group. These results demonstrate the significant influence of P4 on follicular growth.

Another component that is involved in puberty attainment is leptin, a hormone synthesized and released mainly by the adipose tissue (Amstalden et al., 2000). It plays a very important role in this neuroendocrine axis, through two other groups of neurons, neuropeptide Y (NPY), and proopiomelanocortin (POMC), and also acts directly in the anterior pituitary (Williams et al., 2002). While NPY induces negative feedback on GnRH neurons, POMC acts by stimulating these neurons. Therefore, with the body growth of nulliparous animals and fat deposition, there is a stimulus for leptin production and release, which will act by inhibiting NPY neurons and stimulating POMC, thus stimulating the action of GnRH neurons (Alves et al., 2015; Cardoso et al., 2015). The environmental influences, especially nutrition, play an important role in determining when heifers reach puberty. This occurs along with the reduction of negative feedback from E2, which is mediated by a neuronal network responsible for modulating the release of GnRH.

**Figure 1:**
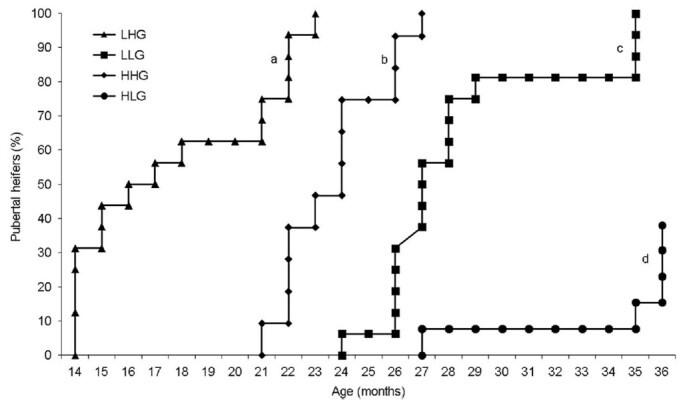
Cumulative proportion (%) of Nelore heifers reaching puberty under different treatments. Treatments included heifers born from sires with varying expected progeny differences (EPD) for age at first calving (L = low; H = high), subjected to either high (HG; 0.7 kg) or low (LG; 0.3 kg) average daily gain. Thus, four treatments were created: LHG (n = 17), LLG (n = 16), HHG (n = 11) and HLG (n = 13). Lines with different superscripts indicate significant differences at P < 0.05. Adapted from Ferraz et al. (2018).

Other factors can interfere with the physiological process of puberty. Genetics, for instance, is of extreme relevance. For example, subspecies differences show that *Bos taurus* heifers generally reach puberty earlier than *Bos indicus* (zebu), which reduce the age at first calving (Nogueira, 2004; Patterson et al., 1992). Moreover, genetics plays an important role in the occurrence of puberty. In this context, an elegant study conducted by Ferraz et al. (2018) factorialized the effects of sire estimated progeny difference (EPD) (either high or low) with the effects of heifer targeted body weight gain (either high or low). Heifers with superior genetic potential for age at first calving (sired by bulls with low EPD for age at first calving) attained puberty sooner only when provided with a diet designed for higher body weight daily gain (Figure 1). Conversely, heifers with high EPD for this trait, when fed a low-gain diet, experienced puberty at a later stage. This investigation underscores the combined influence of genetics and nutrition on the onset of puberty.

**Figure 2:**
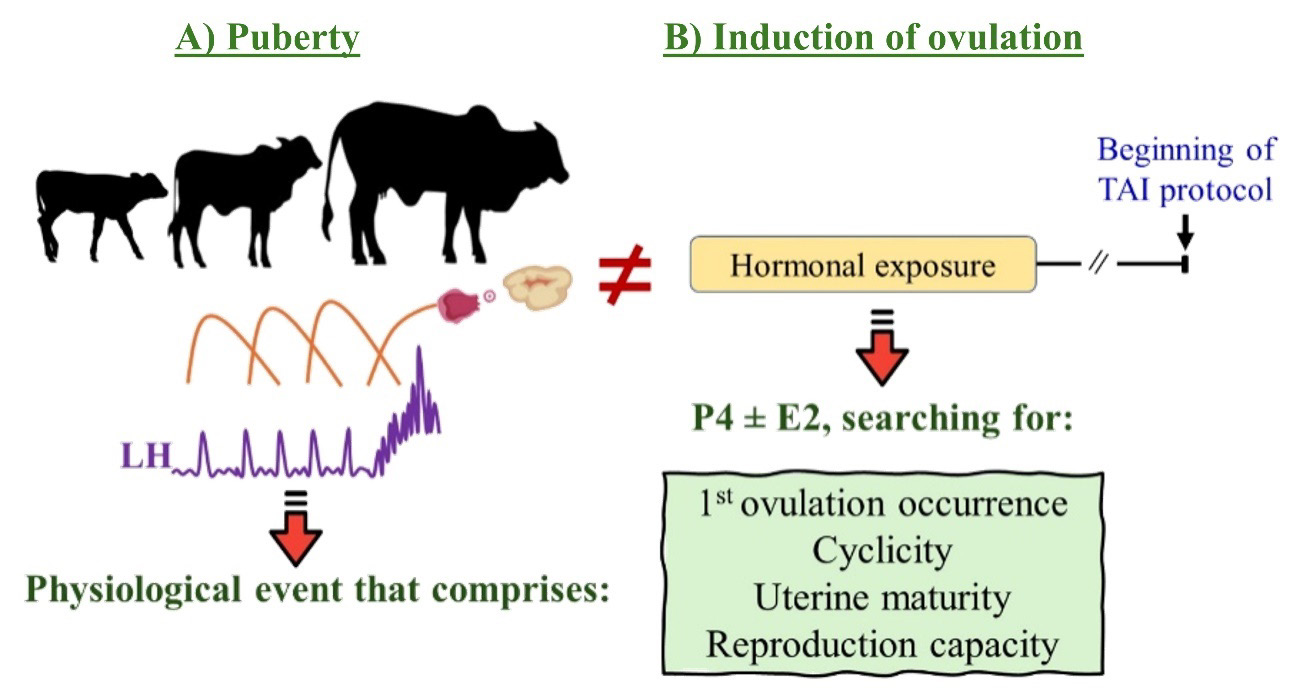
Schematic representation of the physiology underlying puberty and the objectives of an ovulation induction protocol in beef heifers. (A) Puberty encompasses a series of physiological events characterized by gonadotropic and metabolic changes, including the maturation of the HPG axis, increasing GnRH/LH pulsatility. This stimulates follicle development, leading to an E2 peak, culminating in a GnRH/LH surge, the occurrence of the first ovulation, the establishment of cyclicity, uterine maturation, and the attainment of reproductive capacity; (B) Conversely, ovulation induction protocols aim to achieve similar objectives.

The exposure to hormonal protocols, based on progesterone (P4), with or without the addition of other hormones (such as equine chorionic gonadotropin [eCG] and/or E2 esters), can also anticipate the first ovulation and sexual maturity (Anderson et al., 1996; Rodrigues et al., 2013), through stimulation of the HPG axis, and uterine development (Day and Anderson, 1998; Lima et al., 2020). However, inducing puberty differs from inducing ovulation, as induced ovulation does not guarantee that the heifer will continue cycling and be prepared to establish and maintain pregnancy following hormonal exposure (Figure 2).

**Figure 3:**
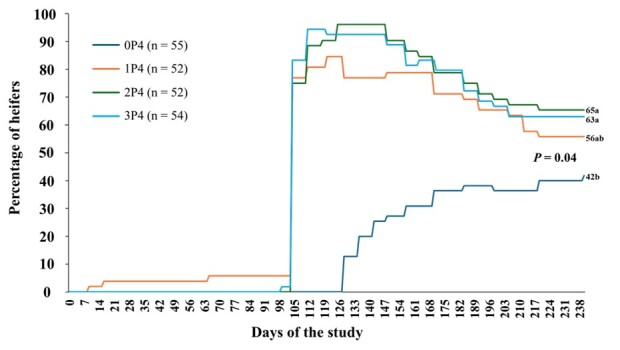
Survival curve of heifers that ovulated and maintained cyclicity under different treatments. The heifers were randomly assigned to four treatments. The control group (0P4) did not receive any hormonal treatment. The group (1P4) followed a 12-d ovulation induction protocol with an intravaginal progesterone (P4) device, followed by estradiol cypionate (EC) administration at P4 device removal. The group (2P4) underwent the same ovulation induction protocol and then a 7-d synchronization protocol. The group (3P4) had three hormonal exposures: one ovulation induction protocol and two synchronization protocols. Adapted from Barroso (2023).

In this sense, Barroso (2023) investigated the cyclicity response of 14-mo old Nelore heifers submitted to hormonal protocols aimed at inducing and/or synchronizing ovulation. The heifers were randomly assigned to four treatments. The control group (0P4) did not receive any hormonal exposure. The group (1P4) underwent a 12-d ovulation induction protocol with an intravaginal P4 device, followed by E2 cypionate (EC) administration at the time of removal. The group (2P4) was subjected to the same ovulation induction protocol, followed by a conventional 7-d synchronization protocol. Finally, the group (3P4) received three hormonal exposures, consisting of one ovulation induction protocol and two synchronization protocols. Results indicated that, despite ovulation occurring in all groups, including spontaneous ovulation in the 0P4, a proportion of heifers returned to the prepubertal condition, without CL (0P4: 12%, 1P4: 37%, 2P4: 32%, and 3P4: 37%), remaining noncyclic thereafter (Figure 3).

In the same way, our group conducted a study to evaluate the reproductive outcomes of 11-mo old Nelore heifers subjected or not to two ovulation induction protocols prior to the TAI protocol (Alves et al., 2023b). The heifers were randomly divided into two groups: control (CON), with no treatment before TAI, and ovulation induction protocol (2IND), receiving two ovulation induction protocols based on P4 plus E2, each lasting 7-d with a 21-d interval between them, before starting the TAI protocol. Subsequently, all heifers received the same TAI protocol. At the onset of the TAI protocol (day 0, time of P4 device insertion), 80.3% (240/299) of induced heifers had CL. However, during the pregnancy diagnosis 30 d after TAI, 56.9% (103/181) of nonpregnant heifers had CL, and 76.2% of these heifers had CL previously, on day 0, indicating that some of them returned to an anestrous state even after undergoing three hormonal treatments (two inductions and the TAI protocol). These results suggest a gap between ovulation induction via hormonal protocols and the achievement of puberty, considering that, upon reaching puberty, these animals begin to exhibit regular cyclicity.

The age at puberty significantly influences the profitability of cattle breeding operations as it directly affects the future productivity of the heifers. Early onset of reproduction is correlated with improved reproductive performance and higher chances of reconception after calving (Funston et al., 2012). In Brazil, where the predominant herd consists of *Bos indicus* females that reach puberty later than *Bos taurus* females, implementing genetic improvement, adequate nutritional management, and modern reproductive strategies alongside with TAI protocols is crucial to render these females reproductively capable. Combining the approaches of genetic improvement, nutritional management and modern reproductive strategies proves to be an effective and feasible tool to be implemented, prolonging the productive life of animals and yielding substantial economic gains for production systems (Núñez-Dominguez et al., 1991; Brumatti et al., 2011; Day and Nogueira, 2013).

## Strategies to induce ovulation prior to TAI protocol

Studies indicated that cyclic beef heifers, characterized by the presence of a CL at the onset of the breeding season, achieved a greater pregnancy per AI (P/AI) compared to noncyclic heifers (Sá et al., 2010). Utilizing tools to increase the proportion of females with a CL at the start of the breeding season, such as hormonal treatments, can be achieved through strategic hormonal strategies aimed at modulating the endocrine environment.

The primary objective of induction of ovulation protocols prior to the TAI protocol is to expedite the onset of reproductive activity in heifers. In pursuit of this goal, some key factors must be considered, as illustrated in Figure 2: uterine maturity, occurrence of first ovulation/cyclicity and reproductive capacity (pregnancy), driven by micro and macroscopic physiological events. Therefore, the following section discusses these critical points, focusing on *Bos indicus* (Nelore) and *Bos taurus*/crossbred heifers.

## Induced ovulation protocols for Nelore heifers

Numerous studies have investigated the effects of hormonal exposure prior to TAI protocols on reproductive outcomes in Nelore heifers. These studies were done with heifers that can be categorized into two groups based on age: regular, typically around 24-mo old, and yearling heifers, comprising heifers younger than 20 mo of age. To better understand these effects, a comprehensive review of the main studies conducted over the past 15 years was performed. The results pertaining to ovulation response and P/AI are summarized in Table 1 (yearling) and Table 2 (regular heifers).

**Table 1:** Overview of studies assessing the impact of hormonal protocols involving progesterone (P4) or P4/estradiol (E2) prior to timed-AI (TAI) protocols on the ovulatory response and fertility in yearling Nelore (*Bos indicus*) heifers (≤ 20 mo old).

**Author**	**n**	**Body weight**	**Pharmacological basis (treated groups)**	**Ovulation response, %**			**P/AI, %**		
				**Control** **1**	**P4** **2**	**P4 + E2** **3**	**Control^1^**	**P4^2^**	**P4 + E2^3^**
Freitas et al. (2015)	626	261.3 ± 0.9	IVDP4 + E2	7.8	-	75.2	42.9	-	43.0
Lemes (2017)	374	241.5 ± 1.5	iP4 + E2 *vs.* IVDP4 + E2	4.9	-	66.0	24.6	-	34.5
Vrisman et al. (2018)	57	289.6 ± 32.3	IVDP4 + GnRH *vs.* GnRH	-	89.7	-	-	-	-
Sampaio et al. (2018)	399	300.0	IVDP4 + E2 *vs.* IVDP4 + PGF	-	-	-	-	-	53.9
Souza et al. (2018)	528	303.1 ± 30.2	IVDP4 + E2 *vs.* iP4 *vs.* iP4 + E2	30.8	56.9	62.2	23.9	40.6	34.9
Magi et al. (2020)	1528	270.0 ± 9.0	iP4 *vs.* IVDP4 + E2	-	-	-	45.5	54.1	53.6
Ferreira et al. (2022)	572	-	iP4 + MGA + E2 *vs.* MGA + E2	-	-	-	-	-	42.1
Silva et al. (2023)	1720	-	iP4	-	-	-	31.1	28.1	-
Alves et al. (2023b)	583	272.0 ± 39.8	IVDP4 + E2	32.4	-	80.3	41.5	-	39.5
Barroso (2023)	213	299.8 ± 5.9	IVDP4 + E2	30.9	-	76.6	-	-	-
Gricio et al. (2023)	638	297.5 ± 1.1	iP4 *vs.* iP4 + E2	-	50.8	62.1	-	47.0	53.3
Lopes et al. (2023)	2300	230.1 ± 0.8	iP4 *vs.* IVDP4 + E2	-	16.1	59.3	-	41.2	47.0
Cavalcanti et al. (2024)	1441	297.8 ± 36.2	IVDP4 + E2	28.8	-	84.3	46.5	-	48.8
Kolling et al. (2024)	683	267.0 ±41.1	IVDP4 + E2	16.6	-	62.2	41. 6	-	35.1
Lanza et al. (2024)	626	276.6 ± 14.9	iP4 *vs.* iP4 + E2 *vs.* IVDP4 + E2	-	47.9	65.1	-	40.0	38.8
**Overall**	-	-	-	**22.7 (403/1776)**	**29.2 (530/1818)**	**69.4 (3065/4418)**	**40.9 (1114/2724)**	**39.3 (1362/3467)**	**45.2 (2608/5770)**

^1^Heifers without hormonal treatments before the initiation of TAI protocols. ^2^Heifers submitted to P4-based protocols before the initiation of TAI protocols. ^3^Heifers submitted to P4/E2-based protocols before the initiation of TAI protocols.

**Table 2:** Overview of studies assessing the impact of hormonal protocols involving progesterone (P4) or P4/estradiol (E2) prior to timed-AI (TAI) protocols on the ovulatory response and fertility in regular Nelore (*Bos indicus*) heifers (> 20 mo old).

**Author**	**n**	**Body weight**	**Pharmacological basis (treated groups)**	**Ovulation response, %**			**P/AI, %**		
				**Control** **1**	**P4** **2**	**P4 + E2** **3**	**Control^1^**	**P4^2^**	**P4 + E2^3^**
Massao et al. (2010)	636	290.0	IVDP4 + E2	35.5	-	57.5	-	-	-
Rodrigues et al. (2013)	1039	294.0 ± 0.8	IVDP4	18.6	39.1	-	-	-	-
Rodrigues et al. (2013)	896	-	IVDP4 *vs.* IVDP4 + eCG	-	62.6	-	-	-	-
Rodrigues et al. (2013)	839	306.0 ± 0.9	IVDP4 *vs.* IVDP4 + eCG *vs.* IVDP4 + eCG + E2	-	65.3	90.1	-	-	-
Rodrigues et al. (2013)	948	327.7 ± 1.1	IVDP4 *vs.* IVDP4 + eCG *vs.* IVDP4 + eCG + E2 *vs.* IVDP4 + E2	-	80.0	82.9	-	-	-
Rodrigues et al. (2014)	774	327.7 ± 1.1	IVDP4	-	88.5	-	-	39.8	-
Sá et al. (2015)	27	-	IVDP4	0.0	42.1	-	-	-	-
Sá et al. (2015)	388	-	IVDP4 + E2	60.3	-	83.0	49.6	-	56.4
Sá et al. (2015)	480	-	IVDP4 + E2	42.5	58.5	65.6	20.2	29.2	31.6
Sá et al. (2015)	640	-	IVDP4 + E2	60.7	-	83.3	26.5	-	43.3
Felisbino et al. (2016)	1174	-	IVDP4 + E2 *vs.* iP4 + E2	-	-	80.5	-	-	43.2
Ferreira et al. (2016)	43	283.0	IVDP4 + E2 *vs.* iP4 + E2	14.0	-	55.2	-	-	-
Gonçalves et al. (2016)	227	250.0	IVDP4 + E2	-	-	67.0	-	-	-
Lima et al. (2020)	638	295.0 ± 24.8	iP4 + E2	11.6	-	63.3	34.2	-	44.7
Santos et al. (2019)	259	335.4 ± 27.7	IVDP4 + E2 *vs.* iP4 + E2 *vs.* iP4	-	80.5	86.6	-	44.8	51.7
Miguez González et al. (2020)	127	318.0 ± 7.0	IVDP4 + E2 *vs.* iP4 + E2	-	-	84.3	-	-	-
Madureira et al. (2020)	828	307.1 ± 22.6	IVDP4 + E2	-	-	78.0		-	54.5
Prata et al. (2020)	742	-	IVDP4 + E2		-	80.1		-	47.3
Machuca et al. (2022)	606	350.9 ± 35.2	iP4 + E2	-	-	45.2	-	-	52.3
Sousa et al. (2022)	258	-	IVDP4 + E2 *vs.* iP4 + E2	-	-	46.9	-	-	47.7
Alves et al. (2024)	769	312.2 ± 0.9	IVDP4 + E2	-	-	84.0	-	-	51.2
**Overall**	-	-	-	**38.5 (396/1029)**	**65.2 (2268/3476)**	**74.4 (5185/6972)**	**31.9 (230/720)**	**38.8 (385/991)**	**48.1 (2678/5573)**

^1^Heifers without hormonal treatments before the initiation of TAI protocols. ^2^Heifers submitted to P4-based protocols before the initiation of TAI protocols. ^3^Heifers submitted to P4/E2-based protocols before the initiation of TAI protocols.

The first objective of the induction protocol is to optimize uterine maturity, as it is directly associated with puberty and improved reproductive performance (Kasimanickam et al., 2020; Monteiro et al., 2013). Macroscopic changes in the uterus are evident when heifers undergo hormonal exposures, particularly with protocols based on P4 alone or in combination with E2. In a study conducted by Claro et al. (2010), 24-mo old prepubertal Nelore heifers exhibited increased uterine scores, characterized by a greater diameter of the uterine horns and increased uterine tone, after receiving intravaginal P4 devices (IVDP4) for 12 d, than heifers that did not receive the IVDP4. Similarly, Lima et al. (2020) investigated the effects of hormonal protocols to induce ovulation prior to a TAI protocol in prepubertal and pubertal Nelore heifers, utilizing injectable P4 (iP4). Their findings indicated a positive impact of P4 on uterine scores in prepubertal females, with scores resembling those of pubertal heifers. Consistent results were reported by Silva et al. (2023), who observed greater uterine scores at the onset of the TAI protocol in 13.5-mo old Nelore heifers following two administrations of iP4, 12 d apart, compared to untreated heifers.

The administration of E2, in conjunction with P4 exposure, has been shown to enhance uterine maturity. Alves et al. (2023b) demonstrated positive effects of P4/E2-based hormonal protocols administered to 11-mo old Nelore heifers prior to the TAI protocol, resulting in an increased uterine diameter at the beginning of the TAI protocol, compared to untreated heifers. Similarly, Kolling et al. (2024) described positive effects of P4/E2-based induction protocols on increasing uterine diameter. Furthermore, Lanza et al. (2024) investigated different ovulation induction strategies in 12-mo old Nelore heifers prior to the TAI protocol. Protocols combining P4, administered by injection or as an IVD, in combination with injectable E2 resulted in significantly greater uterine scores compared to protocols based solely on P4. In terms of fertility, Martins et al. (2017) reported greater P/AI among 14-mo old Nelore heifers with larger uterus, both in natural breeding and TAI programs. Hence, it is imperative to identify hormonal strategies that promote enhanced uterine development in heifers, ultimately resulting in superior reproductive performance.

The second objective of the induction protocol is to stimulate ovulation prior to the initiation of the TAI protocol. This objective is driven by the positive effects of exposure to E2, before ovulation, and P4, released by a CL, after ovulation, as discussed previously in this section.

Several studies have reported a positive effect of P4 exposure on ovulation occurrence in both yearling (Table 1) and regular (Table 2) Nelore heifers. Souza et al. (2018) found a greater incidence of CL at the beginning of the TAI protocol in 14-mo old Nelore heifers treated with iP4 compared to untreated females (56.9% vs. 30.8%). Similarly, Sá et al. (2015) reported greater ovulatory response when 24-mo old Nelore heifers received P4 device for 10 d compared to untreated heifers (58.5 vs. 42.5%). The precise physiological mechanisms underlying the role of P4 in ovulation induction are not fully understood. However, it is well-stablished that low concentrations of P4 increase follicular diameter, leading to elevated circulating E2. This process facilitates GnRH/LH surge after removing the source of P4, resulting in subsequent ovulation (Alves et al., 2023a; Anderson et al., 1996; Day et al., 1987).

The collective findings showed in Table 1 and 2 indicate that P4/E2-based protocols yield greater ovulation response compared to untreated heifers and those exposed solely to P4-based protocols. It is established in the literature that E2, such as benzoate and cypionate, function as ovulation inducers (Sales et al., 2012), thereby explaining the increased ovulation occurrence in heifers treated with E2 esters. In this sense, two studies conducted by our group demonstrated that yearling Nelore heifers subjected to P4/E2-based protocols prior to the TAI protocol exhibited a higher occurrence of ovulation, and thereby, initiating a TAI protocol with CL, compared to untreated heifers (Alves et al., 2023b Kolling et al., 2024).

Some studies have directly compared strategies to induce ovulation using either P4 alone or in combination with E2 administration. Lopes et al. (2023) observed a greater incidence of CL at the beginning of the TAI protocol (day 0) in 12-mo old Nelore heifers treated with an intravaginal P4 device (IVDP4) for 12 d (from days -24 to -12) and E2 administration on day -12, compared to heifers receiving only iP4 on day -24 (59.3 vs. 16.1%). Similarly, Gricio et al. (2023) reported a greater occurrence of CL on day 0 of the TAI protocol in 11.5-mo old Nelore heifers treated with iP4 on day -24 and E2 on day -12, compared to heifers treated only with iP4 on day -24 (62.1 vs. 50.8%). Furthermore, Lanza et al. (2024) conducted an experiment with 12-mo old heifers assigned to four ovulation induction protocols prior to the TAI protocol: two IVDP4/E2-based treatments, one IVDP4/E2-based treatment, one iP4/E2-based treatment, and one iP4-based treatment. The authors observed differences in the presence of CL at the beginning of the TAI protocol, with the lowest percentage in the iP4-based treatment (70.8 vs. 66.5 vs. 57.8 vs. 47.8%, respectively).

Other studies have reported similar results regarding the effectiveness of E2 in increasing ovulatory responses compared to P4 exposure alone in regular heifers. Sá et al. (2015) investigated the ovulatory response in 24-mo old Nelore heifers treated with IVDP4 for 10 d, with or without administration of EC at the time of P4 removal. A higher proportion of heifers receiving EC exhibited CL 30 d after P4 removal compared to those treated solely with P4 (67.2 vs. 58.5%). Similarly, Rodrigues et al. (2013) observed a greater ovulation occurrence in 25-mo old Nelore heifers treated with IVDP4 for 12 d and EC plus eCG administration at the time of device removal, compared to heifers treated only with IVDP4 (85.5 vs. 74.9%). Additionally, Santos et al. (2019) reported higher ovulation incidence in 24-mo old Nelore heifers treated with IVDP4 combined with EC at device removal compared to those receiving either iP4 associated with E2 12 d later or iP4 alone (93.7 vs. 80.1 vs. 80.3%, respectively). Moreover, Sousa et al. (2022) reported greater ovulation occurrence in heifers that received IVDP4, associated with EC at the time of device removal, in comparison to heifers receiving iP4 and EC, 12 d later (54.3 vs. 39.5%). These findings suggest that the combination of P4 and E2 administration may enhance the ovulation response, with IVD as a potentially advantageous source of P4 for achieving higher ovulation rates.

In terms of fertility, the results exhibit substantial variation observed between yearling and regular heifers. Considering yearling heifers (Table 1), Lemes (2017) observed greater P/AI in heifers treated with IVDP4 for 10 d, combined with EC at the time of device removal, compared to untreated heifers and those treated with iP4 and EC administered 10 d later (42.7 vs. 26.5 vs. 24.6%, respectively). Similarly, Lopes et al. (2023) reported improved fertility in heifers treated with IVDP4 (day -24) followed by EC administration 12 d later, compared to those treated solely with iP4 24 d before the onset (day 0) of the TAI protocol (47.0 vs. 41.2%). Gricio et al. (2023) found that EC administration on day -12 enhanced P/AI in heifers previously treated (day -24) with iP4 (53.3 vs. 47.0%), compared to heifers that only received iP4 on day -24. Conversely, Magi et al. (2020) reported higher fertility in heifers treated with either IVDP4 or iP4 (day -24), both combined with EC administered 12 d later, compared to untreated heifers (53.5 vs. 54.0 vs. 45.5%, respectively).

On the other hand, Freitas et al. (2015) did not observe a positive effect on fertility of induction ovulation protocols based on IVDP4 inserted on day -22, with EC administration on day -12 concurrent with device removal, compared to heifers that received no treatment (43.0 vs. 42.9%). Similarly, Lanza et al. (2024) reported no significant difference among strategies for inducing ovulation before initiating the TAI protocol, whether based on IVD P4 plus E2, iP4 plus E2, or iP4 alone. There is clearly an inconsistency in the response results to ovulation induction and fertility protocols. Although the heifers respond to the ovulation induction protocol and reach the TAI protocol with a CL, this does not necessarily mean they have reached puberty and complete sexual maturity. A set of morphophysiological changes, discussed throughout this manuscript, is required to provide the animal with the reproductive competence necessary for conception and pregnancy maintenance.

Considering regular heifers (Table 2), Sá et al. (2015) reported greater P/AI in heifers treated with IVDP4 for 10 d, combined with E2 cypionate (EC) administration at the time of device removal, 30 d before the initiation of TAI protocol, compared to untreated heifers (51.9 vs. 43.6%). Similarly, Lima et al. (2020) described greater P/AI when heifers were submitted to a hormone protocol before the TAI protocol (day 0), involving administration of iP4 on day -22, followed by prostaglandin F-2alpha (PGF) and E2 benzoate (EB) on day -12, compared to untreated heifers (46.0 vs. 38.3%). However, Sousa et al. (2022) reported no significant difference in P/AI between heifers treated with iP4 or IVDP4, both combined with EC 12 d later, which coincided with device removal in the latter group (46.5 vs. 48.8%, respectively).

The benefits on fertility observed in some studies that associated E2 with P4, comparing to untreated heifers or heifers treated exclusively with P4, can be attributed to many aspects. It is well known that E2 directly affect uterine milieu, allowing conception to occur (Bartol et al., 1981; Perry et al., 2005; 2007). Thus, this hormone can exert numerous beneficial effects, spanning from enhancing oocyte quality to optimizing the uterine environment (Binelli et al., 2014; Hawk, 1983; Jinks et al., 2013; Pohler et al., 2012).

In a study conducted by Barroso (2023), sequential hormonal exposures were found to increase cyclicity and uterine maturity of Nelore heifers. Building upon this principle, our research group conducted several studies involving yearling Nelore heifers subjected to two ovulation induction protocols prior to the TAI protocol, utilizing an IVDP4 and EC administration upon withdrawal. These investigations were conducted under diverse management conditions. The first two studies involved heifers housed in a feedlot system with a high-energy (gaining 0.7 to 1.0 kg/d). In the third study, heifers were managed on pasture with supplementation and moderate gain (0.5 kg/d). In the initial study, no difference in P/AI was observed between treated heifers and the untreated group (Alves et al., 2023b). However, in the second study, in which the heifers were younger than those in the first study, hormonal exposure showed a negative effect on P/AI (Kolling et al., 2024). In the third study, heifers subjected to two hormonal exposures demonstrated greater P/AI compared to those in the control group (not exposed to hormonal treatment) or those undergoing only one induction protocol (Cavalcanti et al., 2024). These findings underscore the significant impact of nutrition and genetics on reproductive outcomes in heifers subjected to similar hormonal protocols.

Consistently observed across our latest studies, irrespective of hormonal exposure prior to the TAI protocol, is the beneficial impact of the presence of CL at the beginning of the TAI protocol on reproductive parameters. Table 3 provides a summary of four studies conducted with yearling Nelore heifers under similar conditions (feedlot system). Heifers with a CL on day 0 of the TAI protocol exhibited greater expression of estrus and P/AI at both 30 and 60 d post AI. When categorizing heifers into four groups based on hormonal exposure (untreated vs. treated) and the presence of CL (without vs. with), studies 1 and 3, which included a control group without treatment prior to the TAI protocol, revealed that the highest fertility outcomes were attributed to untreated heifers with CL (i.e. heifers that have probably achieved a natural puberty), while the poorest outcomes were observed in treated heifers without CL. This underscores the importance of genetic selection for precocity and nutritional management, as natural ovulation yielded superior results. Moreover, the absence of ovulation following a hormonal protocol indicates unpreparedness for conception and/or pregnancy maintenance, resulting in unsatisfactory outcomes.

**Table 3:** Expression of estrus and pregnancy per AI (P/AI), 30 and 60 d after AI, in yearling Nelore heifers (*Bos indicus*) with or without corpus luteum (CL) at the onset of a timed-AI (TAI) protocol (day of progesterone device insertion).

**Author**	**n**	**Expression of estrus, %** **1**		**P/AI 30 d, %^1^**		**P/AI 60 d, %^1^**	
		**Without CL**	**With CL**	**Without CL**	**With CL**	**Without CL**	**With CL**
Alves et al. (2023b)	583	78.5	86.4	36.7	43.4	32.3	37.7
Lopes et al. (2023)	2260	58.7	70.0	37.7	54.9	-	-
Kolling et al. (2024)	683	64.1	81.0	31.6	48.9	26.5	46.3
Lanza et al. (2024)	615	-	-	34.6	42.7	32.1	40.3
**Overall**	-	**62.2 (1301/2093)**	**75.9 (1087/1433)**	**36.2 (845/2336)**	**49.4 (891/1805)**	**29.6** **(269/909)**	**41.0** **(399/972)**

^1^All results are different between groups with vs without CL (P ≤ 0.05).

## Induced ovulation protocols for Bos taurus and crossbred heifers

*Bos taurus* and crossbred beef heifers, when properly managed nutritionally, in addition to their genetic potential, typically reach puberty before the first reproductive season (Day and Nogueira, 2013). This fact justifies the scarcity of studies on ovulation induction for these genetic groups. However, there are pharmacological options to anticipate the first ovulation and maximize productive efficiency in beef operations. One of the most common strategies is the administration of exogenous P4, alone or in combination with other hormones (such as E2, eCG, GnRH, or prostaglandins [PGF2α]). With the aim of regulating the HPG axis and stimulating gonadotropin secretion, thus promoting the onset or anticipation of the estrous cycle, several studies, including those by (Patterson et al., 1990; Anderson et al., 1996; Hall et al., 1997; Rasby et al., 1998), have investigated the effects of exogenous sources of P4 on the performance of prepubertal *Bos taurus* and crossbred beef heifers. Their findings indicate that exposure to P4 results in an increase in pulsatile LH release, followed by the manifestation of estrus and, consequently, ovulation. The results related to ovulation response and P/AI are summarized in Table 4 (10 to 24 mo old *Bos taurus* and crossbred beef heifers).

**Table 4:** Overview of studies assessing the impact of hormonal protocols involving progesterone (P4) or P4/estradiol (E2) prior to timed-AI (TAI) protocols on ovulatory response and fertility in *Bos taurus* and crossbred heifers (10 to 24 mo old).

**Author**	**n**	**Body weight**	**Pharmacological basis (treated groups)**	**Ovulation response, %**			**P/AI, %**		
				**Control** **1**	**P4** **2**	**P4 + E2** **3**	**Control^1^**	**P4^2^**	**P4 + E2^3^**
Patterson et al. (1990)	60	261.3 ± 0.9	MGA *vs.* MGA + GnRH	-	48.3	-	-	58.3	-
Anderson et al. (1996)	68	321.5	NORG	0.0	79.4	-	-	-	-
Hall et al. (1997)	68	-	NORG	5.7	30.3	-	-	-	-
Rasby et al. (1998)	311	292 ± 45	IVDP4 *vs.* IVDP4 + E2	37.0	57.8	83.2	-	-	-
Imwalle et al. (1998)	17	284.5	MGA	44.4	100	-	-	-	-
Pfeifer et al. (2009a)	38	287.5	IVDP4 *vs.* IVDP4 + PGF	7.1	50.0	-	-	-	-
Pfeifer et al. (2009b)	40	295.0	IVDP4	-	-	-	36.8	33.3	-
Valentim et al. (2010)	126	270.0	IVDP4 + E2	23.0	-	65.1	-	-	-
Leonardi et al. (2012)	26	250.0	IVDP4 + iP4 + BE + PGF	7.1	-	83.3	-	-	-
Martini et al. (2016)	245	.	MGA + E2 *vs.* IVDP4 + E2	7.7	-	77.2	34.6	-	67.0
Moriel et al. (2017)	120	-	IVDP4 + PGF + GnRH	-	-	.	69.5	66.7	.
Luiz et al. (2018)	99	328 ± 41	iP4 + E2	-	-	64.4	-	-	59.6
Martins et al. (2021)	214	-	IVDP4 + PGF	44.9	62.6	-	29.4	30.5	-
Gularte et al. (2022)	462	270±2.3	iP4	-	-	-	49.4	45.6	-
Gularte et al. (2022)	51	302.1±4.1	iP4	56.2	60.0	-	.	.	-
**Overall**	**-**	**-**	**-**	**22.4 (100/446)**	**58.1 (236/406)**	**74.2 (328/442)**	**48.4 (287/625)**	**45.9 (239/520)**	**59.7 (159/266)**

^1^Heifers without hormonal treatments before the initiation of TAI protocols. ^2^Heifers submitted to P4-based protocols before the initiation of TAI protocol. ^3^Heifers submitted to P4/E2-based protocols before the initiation of TAI protocol.

Consistently, as demonstrated in other studies, P4 supplementation stimulates the development of the uterus in yearling/prepubertal females. For example, findings reported by (Gutierrez et al., 2014) observed a significant increase in uterine diameter after the use of intravaginal P4 devices for 7 d, resulting in greater P/AI, both after natural mating or TAI. It is well established that the addition of E2 favorably modulates the uterine environment, accelerating sexual maturity and resulting in improved reproductive performance. Valentim et al. (2010) investigated and found a positive effect of the P4+E2 combination on uterine development in prepubertal 24-mo old crossbred heifers.

Other studies demonstrate, regardless of breed, the positive effects of presence of CL in heifers at the onset of the TAI protocol, as discussed earlier in this section. Upon receiving an auricular implant containing progestogen (Norgestomet) for 10 d, Hall et al. (1997) found a greater ovulation incidence in crossbred beef heifers compared to those that did not receive any treatment (30.3 vs. 5.7%). Anderson et al. (1996), following the same methodology for inducing cyclicity, found similar results for the control and treated groups (0.0 vs. 79.4%). The lack of ovulation response can be attributed to the absence of P4 in the control group, which also plays an important role in the regulation of the reproductive axis in *Bos taurus* prepubertal heifers. It acts by reducing the expression of E2 receptors at the hypothalamic level, decreasing the negative feedback on GnRH, resulting in increased LH pulsatility, essential for triggering ovulation (Day et al., 1984; Day et al., 1987). Imwalle et al. (1998) again reported the effectiveness of an exogenous source of P4 in increasing the incidence of ovulation in heifers.

These primary studies encouraged other researchers to conduct additional investigations, aiming to enhance the response to these protocols. Valentim et al. (2010), found in their study a higher percentage of heifers with CL after exposure to P4/E2 compared to those that did not receive prior treatment (65.1 vs. 23.0%). Similarly, Leonardi et al. (2012) found a significant difference in ovulation of heifers exposed to protocols with the combination of P4/E2 compared to controls (83.3 vs. 7.1%). Martini et al. (2016) demonstrated a higher response for the P4/E2 group compared to controls (77.2 vs. 7.7%). These studies consistently corroborate data addressed in the literature regarding the beneficial effect of adding an E2 ester to an ovulation induction protocol.

For *Bos taurus* and crossbred beef heifers, the variability found in fertility results is repeated when compared to Nelore. Furthermore, few studies have evaluated fertility by comparing untreated groups with those exposed to induction protocols beforehand. Martins et al. (2021) found no significant differences in P/AI when treating heifers with IVDP4 or nothing (30.5 vs. 29.4%), respectively. Pfeifer et al. (2009b) and Gularte et al. (2022) also found no results indicating that exposure to P4 increased P/AI (33.3 vs. 36.8; 45.6 vs. 49.4%) compared to untreated groups. In studies that exposed heifers to strategies combining P4/E2, a greater P/AI was observed, however, without comparative experimental groups (Luiz et al. 2018). On the other hand, Martini et al. (2016) observed favorable results regarding fertility when inducing ovulation previously to TAI protocols in heifers with P4/E2 compared to untreated ones, both in terms of P/AI and final pregnancy rate (59.8 vs. 34.6; 74.2 vs. 55.0%), respectively. These results may be attributed to the greater incidence of CL at the beginning of the TAI protocol for the treated group, as mentioned above. Additionally, improved P/AI may be correlated to the addition of E2 to the induction protocol, considering all the beneficial effects of this hormone on the uterine environment.

## Final considerations

Puberty in *Bos indicus* and *Bos taurus* heifers involves physiological and metabolic changes that lead to sexual maturity and regular cycles. In contrast, hormonal induction of ovulation advances the first ovulation but does not ensure regular cycles. Heifers with a CL before the TAI protocol show greater estrus expression and P/AI compared to those without a CL. The combination of P4 and E2 significantly impacts uterine development, increasing reproductive efficiency. Exposure to P4 has a positive effect on ovulation induction, and the addition of E2 apparently increases the ovulation rate. Overall, studies have shown that fertility varied according to the type of ovulation induction protocol used, with inconsistent results. This highlights the need for further studies to understand and refine these protocols, aiming to increase the efficiency of beef cattle production systems.
